# Pancreatoduodenectomy with vascular reconstruction versus chemotherapy alone in patients with locally advanced pancreatic cancer: a systematic review

**DOI:** 10.1590/0102-67202025000021e1890

**Published:** 2025-08-04

**Authors:** Adriano Carneiro da COSTA, Vitoria Alessandra DUARTE, Fernando SANTA CRUZ, Mohamed Ali CHAOUCH, Jayant KUMAR, Isabella RECCIA, Álvaro Antonio Bandeira FERRAZ, Nagy HABIB

**Affiliations:** 1 Universidade Federal de Pernambuco, Department of Surgery – Recife (PE), Brazil.; 2 University of Monastir, Department of Visceral and Digestive Surgery – Monastir, Tunisia.; 3 Imperial College London, Department of Surgery and Cancer – London, England.

**Keywords:** Pancreatic Neoplasms, Pancreas, Chemotherapy Adjuvant, Pancreaticoduodenectomy, Neoplasias Pancreáticas, Pâncreas, Quimioterapia Combinada, Pancreaticoduodenectomia

## Abstract

**Background:**

Pancreatic cancer is still a terrifying condition that has a high mortality rate due to its rapid progression and treatment complexity. However, there is still no consensus on what the gold standard of treatment for locally advanced pancreatic cancer (LAPC) is.

**Aims:**

The aim of this study was to review the current evidence-based data on treatment strategies for LAPC, comparing pancreatoduodenectomy with vascular reconstruction (PDVR) and chemotherapy alone (CA).

**Methods:**

This systematic review was performed according to the PRISMA 2020 guidelines. Overall survival (OS) was the primary endpoint, while progression-free survival (PFS) was the secondary endpoint. The included studies were published between 2013 and 2023.

**Results:**

A total of 16 relevant papers were found in the literature search. The median PFS duration for CA varied from 3.22 to 11.7 months, whereas the median overall survival (mOS) varied from 5.95 to 23.0 months. The mOS ranged from 12.7 to 24.9 months and the median PFS time ranged from 8.5 to 22.5 months for patients submitted to neoadjuvant therapy followed by PDVR.

**Conclusions:**

LAPC presents worse outcomes when patients are submitted to CA with gemcitabine only, or when patients undergo upfront PDVR.

## ARTICLE HIGHLIGHTS

- Locally advanced pancreatic cancer is a challenging disease that requires a multidisciplinary approach to achieve acceptable results.
- Surgery alone did not show to be an interesting approach. Chemotherapy alone with gemcitabine only also showed worse outcomes.
- When feasible, the combination of multiple drugs associated with surgical resection might be the preferential approach.

## CENTRAL MESSAGE

Locally advanced pancreatic cancer (LAPC) is an unresectable condition without metastases that has venous involvement of the superior mesenteric vein, portal vein, and/or hepatic artery. However, it is not a contraindication for surgery, and initial resection is considered not beneficial due to extensive vascular involvement and consequently a high chance of nonradical resection. LAPC represents approximately 30% of pancreatic cancers, and its prognosis is between that of metastatic and resectable pancreatic cancers.

## PERSPECTIVES

This systematic review demonstrated that for patients with locally advanced pancreatic cancer, the worst outcomes were those who underwent chemotherapy alone with gemcitabine alone and patients who underwent pancreatoduodenectomy surgery with vascular reconstruction without neoadjuvant chemotherapy. On the other hand, patients with the best overall survival were those who received a combined neoadjuvant regimen (fluorouracil, irinotecan, oxaliplatin, and folinic acid [FOLFIRINOX] or nab-paclitaxel [GnP]) followed by surgical resection.

## VISUAL ABSTRACT

**Figure:**
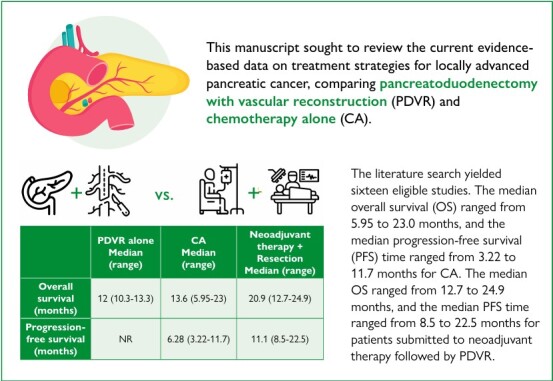

## RESUMO

**Racional:**

O câncer de pâncreas ainda é uma condição assustadora que tem uma taxa de mortalidade elevada devido à sua rápida progressão e complexidade de tratamento. No entanto, ainda não há consenso sobre qual é o padrão ouro de tratamento para câncer de pâncreas localmente avançado (CPLA).

**Objetivos:**

Revisar os dados atuais baseados em evidências sobre estratégias de tratamento para câncer de pâncreas localmente avançado, comparando pancreatoduodenectomia com reconstrução vascular (PDRV) e quimioterapia isolada (QI).

**Métodos:**

Esta revisão sistemática foi realizada de acordo com as diretrizes PRISMA 2020. A sobrevida global (SG) foi o desfecho primário, enquanto a sobrevida livre de progressão (SLP) foi o desfecho secundário. Os estudos incluídos foram publicados entre 2013 e 2023.

**Resultados:**

Dezesseis artigos relevantes foram encontrados na busca bibliográfica. A duração mediana da sobrevida livre de progressão para quimioterapia isolada variou de 3,22 a 11,7 meses, enquanto a sobrevida mediana da sobrevida global variou de 5,95 a 23,0 meses. A mediana de sobrevida global variou de 12,7 a 24,9 meses, e o tempo mediano de sobrevida livre de progressão variou de 8,5 a 22,5 meses para pacientes submetidos à terapia neoadjuvante seguida de pancreatoduodenectomia com reconstrução vascular.

**Conclusões:**

O câncer de pâncreas localmente avançado apresenta piores resultados quando os pacientes são submetidos à quimioterapia apenas com gemcitabina, ou quando os pacientes são submetidos a DP inicial com reconstrução vascular.

## INTRODUCTION

Locally advanced pancreatic cancer (LAPC) is an unresectable condition without metastases that has venous involvement of the superior mesenteric vein (SMV), portal vein (PV), and/or hepatic artery^12^. However, it is not a contraindication for surgery, and initial resection is considered not beneficial due to extensive vascular involvement and consequently a high chance of nonradical resection^17,20^. LAPC represents approximately 30% of pancreatic cancers, and its prognosis is between that of metastatic and resectable pancreatic cancers^15^.

 The surgical excision of tumors (*en bloc* resection of the pancreas and surrounding structures) is still considered the sole possible clinical option for treating pancreatic cancer in individuals with resectable tumors^1,16^. However, treatment for LAPC is still controversial^16^. Historically, resection was deemed contraindicated when neighboring vasculature was involved^14^. However, venous resection with reconstruction during pancreatoduodenectomy is currently performed in up to 20–25% of patients in some centers^8,22^. Despite being increasingly common, vascular resection (VR) during pancreatic surgery is nonstandardized^7^. 

 For pancreatic cancer, total tumor excision is thought to be the only viable treatment^11^. On the other hand, pancreatic tumors are close to nearby blood arteries and tend to invade them. Vascular repair may be necessary for the whole resection of locally advanced pancreatic cancers (LAPCs). Given the higher frequency of problems, the use of pancreaticoduodenectomy with vascular reconstruction (PDVR) is still up for discussion^14^. Prior multi-institutional research has shown that patients who underwent PDVR experienced higher rates of complications and death than individuals who underwent pancreatoduodenectomy (PD) alone^5,26^. 

 The classic first-line treatment for LAPC consists of adjuvant chemotherapy or induction chemotherapy for tumor downsizing, and the most consolidated therapies are FOLFIRINOX (fluorouracil, irinotecan, oxaliplatin, and folinic acid), gemcitabine (Gem), and nab-paclitaxel (GnP) plus Gem^17,19,22-25^. Recent studies have indicated that neoadjuvant therapy (NT) with FOLFIRINOX and GnP combined with Gem increases the conversion rate of tumors previously classified as LAPC and unresectable^2,4,24,28^. 

 To date, the literature on therapies for LAPC and their impact on the survival of affected patients is scarce, with even fewer studies evaluating alternatives to resection associated with vascular reconstruction^7,9,14^. The aim of this systematic review was to analyze the currently available information on existing therapies for LAPC and their impacts on progression-free survival (PFS) and overall survival (OS). 

## METHODS

 The PRISMA 2020 recommendations (1) and AMSTAR 2 guidelines (Assessing the Methodological Quality of Systematic Reviews) (2) were followed in the execution of this systematic review. The protocol was registered under CRD 42022379880 in PROSPERO. 

### Electronics searches

A literature search was performed to assess the currently available information on existing therapies for LAPC and their impacts on PFS and OS. Articles published in the English language, from 2013 to 2023, were retained. The search strategy using a prospectively defined algorithm in PubMed was conducted on August 8, 2023. MeSH terms included (vascular reconstruction) AND (locally advanced pancreatic cancer) AND (chemotherapy), (resection and reconstruction) AND (locally advanced pancreatic cancer) AND (chemotherapy), (resection and reconstruction) AND (locally advanced pancreatic cancer) AND (chemotherapy), (FOLFIRINOX) AND (locally advanced pancreatic cancer) AND (chemotherapy), (FOLFIRINOX) AND (locally advanced pancreatic cancer) AND (chemotherapy). MeSH terms were matched with the following keywords: ("vascular reconstruction" OR "locally advanced pancreatic cancer" OR "chemotherapy" OR "FOLFIRINOX" OR). These terms were combined using Boolean operators ("AND," "OR") to refine the search. A manual search of the reference lists of relevant articles was also carried out. The search yielded 1,168 references. After removing duplicate records and screening titles and abstracts, 422 articles were selected. A total of 406 articles were excluded due to study characteristics or study methodology, and 16 articles were ultimately included in the analysis.

### Inclusion and exclusion criteria

We retained randomized clinical trials and controlled clinical trials that included adult patients who underwent resection and vascular reconstruction versus palliative chemotherapy for LAPC. Only articles published in peer-reviewed journals were considered..

### Outcome measures

OS was the primary endpoint, and PFS was the secondary endpoint.

### Extraction of data

Two authors extracted the data independently, and the senior author resolved any discrepancies through consensus. In this analysis, we chose publications that examined the effects of treatments on PFS and OS in patients treated with vascular reconstruction and chemotherapy for LAPC, as well as patients who underwent resection and Whipple surgery.

## RESULTS

**Figure 1:**
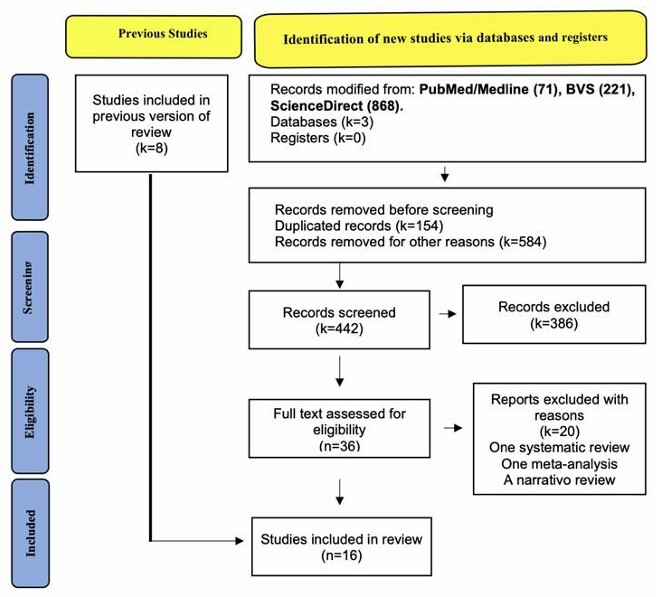
Flowchart for study selection according to the PRISMA 2020 recommendations.

 The literature search yielded 16 eligible studies (Figure 1). A total of eight studies were included in the previous version of the review. A total of 20 studies were excluded: eight systematic reviews and three narrative reviews. A total of four studies involved PDVR^2,9,17,19^, six studies involved chemotherapy alone (CA)^3,6,12,14,15,20^, and four studies involved NT followed by resection^4,13,18,21,22^. There were 5,488 patients in the PDVR group and 3,359 patients in the CA group (Table 1). The median PFS duration for CA was 3.22–11.7 months, and the median overall survival (mOS) varied from 5.95 to 23.0 months. The mOS ranged from 12.7 to 24.9 months, and the median PFS time ranged from 8.5 to 22.5 months for patients submitted to NT followed by resection. The results regarding PDVR alone showed a mOS of 12 months (10.3–13.3 months) (Table 2). The studies were published from 2013 to 2023. A total of five studies were conducted in the USA, three in the Netherlands, two in France, two in Japan, one in Korea, one in Australia, and one in Turkey. The data of the retained studies are presented in Figure 1 and Table 1. 

**Table 1:** Demographic table of patients included in the study.

Authors	Study Design	Country	N	Treatment	Type of treatment	Median PFS (months)	Median OS (months)
Ozaka et al.^17^	Prospective	Japan	125	Chemoterapy alone	Arm A (n=63): GnP	9.4	21.3
					Arm B (n=62): FFX	11.2	23
Santucci et al.^19^	Retrospective	Australia	615	Chemotherapy alone	Arm A (n=376): GnP	5.7	11.3
					Arm B (n=73): FFX	5.1	12.3
Brada et al.^3^	Prospective	Netherlands	293	Both	Arm A (n=89): FFX neo+resection	----	24
					Arm B (n=204): FFX	----	15
Walma et al.^24^	Prospective	Netherlands	422	Both	Arm A (n=32): FFX neo+resection	----	23
					Arm B (n=252): FFX	----	14
					Arm D (n=33): GnP	----	9
					Arm C (n=41): Gem	----	9
Williet et al.^25^	Retrospective	France	147	Chemotherapy alone	Arm A (n=87): FFX	----	12.1
					Arm C (n=60): GnP	----	9
Brada et al.^4^	Prospective	Netherlands	252	Both	Arm A (n=32): FFX neo+resection	----	23
					Arm B (n=220): FFX	----	14
Yalcin et al.^27^	Prospective	Turkey	23	Chemotherapy alone	Arm A (n=12): Gem	3.22	5.95
					Arm B (n=11): GnP	6.28	9.92
Wainberg et al.^23^	Prospective	USA	50	Chemotherapy alone	GnP	5.5	9.9
Yoo et al.^28^	Retrospective	Korea	124	PDVR	FFX neo+resection	11.1	18.4
Shaib et al.^20^	Prospective	USA	16	PDVR	FFX neo+resection or GnP neo+resection	-------	12.7
Matsumoto et al.^16^	Prospective	Japan	66	Chemotherapy alone	FFX	7.6	18.5
Kluger et al.^13^	Prospective	USA	30	PDVR	FFX or GnP neo+resection	8.5	18.5
Dua et al.^7^	Retrospective	USA	90	PDVR	Resection without chemotherapy	------	10.3
Marthey et al.^15^	Prospective	France	77	Both	Arm A (n=49): FFX	11.7	15.9
					Arm B (n=28): FFX neo+resection	22.5	24.9
Gong et al.^9^	Retrospective	China	119	PDVR	Resection without chemotherapy	------	13.3

PFS: progression-free survival; OS: overall survival; GnP: gemcitabine plus nab-Paclitaxel; FFX: FOLFIRINOX; Gem: gemcitabine; PDVR: pancreatoduodenectomy with vascular reconstruction.

**Table 2:** Data regarding overall survival and progression-free survival of the included studies.

	PDVR alone Median (range)	CA Median (range)	NT+PDVR Median (range)
OS	12 (10.3–13.3)	13.6 (5.95–23)	20.9 (12.7–24.9)
PFS	NR	6.9 (3.22–11.7)	12.4 (8.5–22.5)

PDVR: pancreatoduodenectomy with vascular reconstruction; CA: chemotherapy alone; NT: neoadjuvant therapy; OS: overall survival; PFS: progression-free survival.

## DISCUSSION

 This systematic review was performed to study the currently available information on existing therapies for LAPC and their impacts on OS and PFS. Currently, there are a variety of multimodal treatment strategies for resectable adenocarcinoma, with surgery integrated into this multimodal system, including neoadjuvant chemotherapy versus upfront surgery and adjuvant chemotherapy. However, treatment paradigms for LAPC after first-line treatment are lacking. Furthermore, the therapeutic outcome remains subpar, drawing the interest of a growing number of academics globally. Thus, we reviewed the current literature to develop future perspectives on the best treatment option for LAPC. 

 Castleberry et al. investigated the impact of VR on early postoperative outcomes after pancreaticoduodenectomy^5^. The relationship between VR during PD and 30-day postoperative mortality and morbidity was investigated using a retrospective cohort analysis. The analysis comprised 3,582 patients in total, of whom 281 (7.8%) had vascular tissue Whipple surgery. This study showed that patients who underwent PDVR had higher 30-day postoperative morbidity and mortality than patients who underwent PD alone. 

 Worni et al. studied short-term differences among patients who underwent pancreatic resection with and without VR, including 4,022 individuals (4.0%) out of 10,206 patients who received pancreatic resection combined with VR. Patients who underwent pancreatic surgery using virtual reality (VRL) had far higher rates of postoperative complications and mortality than patients without VRL among the 25% of hospitals with the highest surgical volume. This study showed that compared to individuals who underwent pancreatic resection alone, those who was submitted to VR during pancreatic surgery experienced higher rates of unfavorable postoperative outcomes^26^. 

 Many methods have been reported for the reconstruction or excision of the SMV and/or PV during pancreatectomy; however, the best approach is still unknown. Resection of pancreatic tumors involving SMV-PV can be accomplished in places with sufficient experience with respectable morbidity and fatality rates^7^. Lim et al. found no statistically significant variation in the overall rate of morbidity or death after pancreaticoduodenectomy between resection including arterial structures and vein-only resection^14^. On the other hand, following PV/SMV resection, primary end-to-end vascular anastomosis and transverse venorrhaphy should be chosen over patch venoplasty due to their higher patency and lower thrombosis rates^7^. 

 Combination chemotherapy treatments, like Gem with GnP and FOLFIRINOX, have shown a significant survival advantage over Gem alone when treating metastatic pancreatic cancer^17^. Moreover, FOLFIRINOX and GnP have been used in LAPC because of their efficacy. The use of FOLFIRINOX for LAPC is of particular interest given its high response rate of 32% in patients with metastatic disease^16^. FOLFIRINOX-treated patients with LAPC had a median survival of 24.2 months according to a meta-analysis published by Suker et al^21^. 

 FOLFIRINOX dramatically increased OS in a Conroy et al.’s phase III study^6^, and it is currently the accepted treatment modality for metastatic pancreatic cancer. However, treatment for LAPC is still controversial^16^. There is growing evidence that FOLFIRINOX and GnP are effective first-line treatments for patients with LAPC; however, there is still disagreement on this issue^25^. 

 Recently, Osaka et al.^17^ and Santucci et al.^19^ conducted studies on LAPC and compared the efficacy of FOLFIRINOX with that of GnP. Ozaka et al.^17^, through a randomized phase II study, reported a mOS time of 23 versus 21.3 months and a median PFS time of 11.2 versus 9.4 months for FOLFIRINOX and GnP, respectively. According to Santucci et al.^19^, FOLFIRINOX and Gem/Nab-P showed higher efficacy than Gem alone (mOS: 8.9 months) and had comparable outcomes (mOS: 11.4 versus 13.2 months, respectively) in patients with LAPC. 

 Strategies for treating LAPC were studied by Walma et al.^24^. Of the total number of patients (422), 326 (77%) had chemotherapy. Notably, 77% of the patients (252/326) were among those who began FOLFIRINOX. Notably, 10% of the patients (33/326) received GnP, while 13% (41/326) received Gem monotherapy. 

 The mOS of this entire cohort was 10 months. In patients treated with FOLFIRINOX, Gem monotherapy, or GnP, the mOS was 14, 8, and 9 months, respectively. A mOS of 23 months was achieved after resection in 13% (32/252) of patients following FOLFIRINOX. 

 Yoo et al.^28^ investigated the clinical outcomes of neoadjuvant FOLFIRINOX in patients with LAPC and reported that patients who underwent NT and surgery had a mOS of 17.1 months, a median PFS of 10.1 months, and a rate of conversion surgery of 29.0%. In this study, neoadjuvant FOLFIRINOX did not increase postoperative complications in patients with LAPC. Brada et al.^3^ reported that patients who underwent tumor resection after four cycles of induction FOLFIRINOX had a mOS of 23.4 months, while patients who did not undergo tumor resection had a mOS of 13.3 months. These studies indicate that patients with LAPC should receive newer chemotherapy regimens as first-line treatments, particularly FOLFIRINOX, because of a prospective rise in OS. 

 Debatable has been the usefulness of neoadjuvant treatments for pancreatic cancer that is borderline resectable and LAPC^14^, and studies on conversion surgery following FOLFIRINOX have been published with favorable results^10,18^. Since the advent of FOLFIRINOX, an increasing number of cohort studies have indicated favorable results for patients undergoing resection after chemotherapy, with a mOS ranging from 22 to 35 months and a resection rate of 28%^24^. There is increasing evidence that FOLFIRINOX and GnP are first-line therapies for patients with LAPC, resulting in improved survival and resection of unresectable tumors^25^. In the real world, the efficacy of neoadjuvant FOLFIRINOX and surgery appears to be superior to that of FOLFIRINOX and Gem/NabP palliative for LAPC, despite some studies reporting similar results in their respective clinical trials^6^. 

 This study has several limitations. First, there are only a few studies in the literature comparing VR and reconstruction versus palliative chemotherapy for LAPC. Second, only a few variables were analyzed in these studies, with only OS and PFS being described. 

## CONCLUSIONS

This systematic review demonstrated that for patients with LAPC, the worst outcomes were those who underwent CA with Gem alone and patients who underwent PDVR without neoadjuvant chemotherapy. On the other hand, patients with the best OS were those who received a combined neoadjuvant regimen (FOLFIRINOX or GnP) followed by surgical resection.

## Data Availability

The datasets generated and/or analyzed during the current study are available from the corresponding author upon reasonable request.
